# Sex-Dependent Effects of Developmental Lead Exposure in Wistar Rats: Evidence from Behavioral and Molecular Correlates

**DOI:** 10.3390/ijms21082664

**Published:** 2020-04-11

**Authors:** Anna Maria Tartaglione, Melania Maria Serafini, Andrea Raggi, Francesca Iacoponi, Elisa Zianni, Alessandro Scalfari, Luisa Minghetti, Laura Ricceri, Francesco Cubadda, Gemma Calamandrei, Barbara Viviani

**Affiliations:** 1 Centre for Behavioral Sciences and Mental Health, Istituto Superiore di Sanità (ISS), 00161 Rome, Italy; annamaria.tartaglione@iss.it (A.M.T.); alessandro.scalfari@gmail.com (A.S.); laura.ricceri@iss.it (L.R.); 2 Department of Pharmacological and Biomolecular Sciences, Università degli Studi di Milano, 20133 Milan, Italy; melania.serafini@unimi.it (M.M.S.); elisa.zianni@unimi.it (E.Z.); barbara.viviani@unimi.it (B.V.); 3 Department of Food Safety, Nutrition, and Veterinary Public Health, Istituto Superiore di Sanità (ISS), 00161 Rome, Italy; andrea.raggi@iss.it (A.R.); francesca.iacoponi@iss.it (F.I.); francesco.cubadda@iss.it (F.C.); 4 Research Coordination and Support Service, Istituto Superiore di Sanità (ISS), 00161 Rome, Italy; luisa.minghetti@iss.it

**Keywords:** lead, neurodevelopment, neuroplasticity, sexual dimorphism, behavior, glutamatergic receptors

## Abstract

Lead (Pb) exposure in early life affects brain development resulting in cognitive and behavioral deficits. Epidemiologic and experimental evidence of sex as an effect modifier of developmental Pb exposure is emerging. In the present study, we investigated Pb effects on behavior and mechanisms of neuroplasticity in the hippocampus and potential sex differences. To this aim, dams were exposed, from one month pre-mating to offspring weaning, to Pb via drinking water at 5 mg/kg body weight per day. In the offspring of both sexes, the longitudinal assessment of motor, emotional, and cognitive end points was performed. We also evaluated the expression and synaptic distribution of N-methyl-D-Aspartate receptor (NMDA) and α-amino-3-hydroxy-5-methyl-4-isoxazolepropionic acid (AMPA) receptor subunits at post-natal day (pnd) 23 and 70 in the hippocampus. Neonatal motor patterns and explorative behavior in offspring were affected in both sexes. Pb effects in emotional response and memory retention were observed in adult females only, preceded by increased levels of GluN2A and GluA1 subunits at the post-synapse at pnd 23. These data suggest that Pb exposure during development affects glutamatergic receptors distribution at the post-synaptic spine in females. These effects may contribute to alterations in selected behavioral domains.

## 1. Introduction

Early-life exposure to environmental chemicals interferes with developmental programming and induces subclinical alterations that may result in pathophysiology and behavioral deficits later in life, consistently with the Developmental Origins of Health and Disease (DOHaD) hypothesis [1,2,3].

Lead (Pb) is a systemic toxicant primarily affecting the central nervous system (CNS). Exposure of the general population predominantly occurs via the diet, whereas workplace exposure is of concern in specific occupational settings [4,5,6]. Pregnancy and childhood are critical time windows for Pb toxic effects. The metal easily crosses the placenta [7,8] and exhibits a higher gastrointestinal absorption in children compared to adults, which in combination to children behavior (e.g., hand-to-mouth activities) results in higher internal exposures [9].

Biomonitoring studies show a marked decrease in blood levels as a result of control measures taken to regulate lead in paint, petrol, food cans, and pipes since the 1970s [10,11]. However, epidemiological and experimental studies concurrently demonstrated that Pb adverse effects occur at lower exposures than previously thought [12,13,14,15]. As a result, blood Pb level considered of concern for neurodevelopmental toxicity in US children decreased from 60 µg/dL in the 1960s to 10 µg/dL in 1991, and 5 µg/dL in 2012. Current evidence indicates neurological as well as neuropsychological effects even at lower levels and it is now widely recognized that there is no “safe” threshold for developmental neurotoxicity in young children [15,16,17]. Furthermore, Pb shows a non-linear dose-response effect suggesting a greater impact at lower Pb concentrations in infants and children [13,18]. The European Food Safety Authority (EFSA) established a 95th percentile lower confidence limit of the benchmark dose of 1% extra risk (BMDL_01_) of 12 μg blood Pb/L (corresponding to a dietary intake value of 0.50 μg/kg body weight, b.w. per day) as the reference point for intellectual deficits in children measured by the Full Scale IQ score and concluded that there is concern at current levels of Pb exposure for effects on neurodevelopment [19]. In spite of all measures undertaken to reduce environmental sources of exposure, Pb continues to be a public health concern worldwide [20].

Early life chronic Pb exposure induces deleterious effects on neurodevelopmental trajectories and neuronal signaling and plasticity, which in turn accounts for cognitive and behavioral deficits persisting even later in adulthood [14,21].

Different studies point to the involvement of N-methyl-D-Aspartate receptor (NMDAR) expression and function, primarily in the hippocampus, among the mechanisms underlying Pb effects. Patch-clamp recordings from hippocampal neurons showed a blocking effect of Pb on the NMDAR [22,23]. In addition, Pb exposure differently modifies the expression of GluN2A, GluN2B, and GluN1 subunits of the NMDAR [21]. However, the molecular basis of these processes needs to be further investigated.

NMDARs are glutamate-gated ion channels and essential mediators of brain plasticity; they are strongly involved in synaptic structure and functions, and are thought to underlie higher cognitive functions [24]. Several subtypes of NMDARs exist as hetero-tetrameric complexes that associate GluN1 subunits with GluN2 (GluN2A-2D) or a mixture of GluN2 and GluN3 (GluN3A and B) subunits. The differential combination among subunits allows a large repertoire of NMDARs characterized by different biophysical and pharmacological properties [24,25,26]. In the adult hippocampus, GluN2A and GluN2B are the predominant subunits [27,28] and their expression is strictly regulated along with brain development [28]. GluN2B is highly expressed in the developing hippocampus, while GluN2A expression starts after birth, to become highly represented in the adult CNS. This precise regulation is fundamental for excitatory synapse maturation, synaptic connectivity and acquisition of learning abilities [29,30,31]. Any NMDAR dysfunction due to altered expression, localization, or activity may contribute to neurological dysfunctions [32,33,34], as such NMDAR organization across development represents a relevant endpoint of neurotoxicity.

Recent evidence in both humans and animal models suggests that sex might be an effect modifier of developmental Pb exposure [35,36]. Currently available data are not consistent across studies. Only a limited number of epidemiological studies considered gender as a factor in independent outcome analyses whereas many experimental studies did not take into account the sex of the animals or considered only one sex (mainly males).

In particular, experimental studies based on low Pb concentrations mirroring human exposure scenarios, assessing simultaneously brain molecular and behavioral outcomes in the two sexes are surprisingly lacking.

To address these knowledge gaps, in the present study we investigated the potential contribution of sex to Pb effects on behavior and neuroplasticity in the hippocampus, a brain region previously shown to be particularly sensitive to developmental Pb exposure [37].

The aims of this work were: (i) assessing the effects of developmental exposure to a Pb concentration among the lowest used in rodent studies, on a wide range of motor, emotional, and cognitive functions at different age points; (ii) evaluating behavioral sex dimorphism throughout the life course; and (iii) investigating the expression and synaptic localization of ionotropic-glutamatergic receptors (NMDA and AMPA) subunits and their potential role in sex dependent vulnerability to developmental Pb exposure.

**Figure 1:**
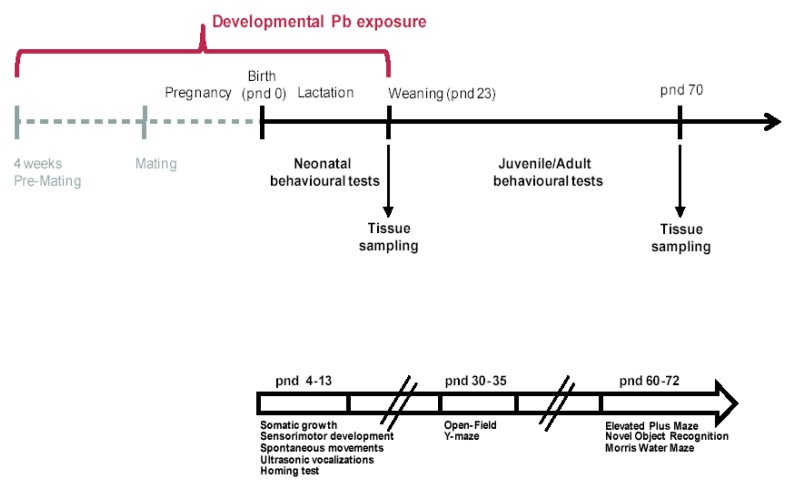
Timeline of the Experimental Design. Developmental lead (Pb) exposure (from 4 weeks pre-mating to offspring weaning), behavioral testing (neonatal and juvenile/adult tests) and tissue collection for Pb determination, in blood and brain, and Western blot on hippocampus at post-natal day 23 and 70 are indicated.

The timeline of experimental design is reported in Figure 1.

## 2. Results

### 2.1. Reproductive Performances of Dams Exposed to Pb

Data collected at birth did not show overt detrimental effects of gestational Pb exposure on number of delivered pups [Mean ± SD, Veh = 15 ± 3.4; Pb = 13 ± 3.0, F (1, 13) = 1.39 *p* = 0.25], sex ratio [Mean ± SD, Veh = 0.67 ± 0.2; Pb = 0.73 ± 0.3, F (1, 13) = 0.126 *p* = 0.72], and body weight of pups at birth [Mean ± SD, Veh = 6.57 ± 1.4; Pb = 6.59 ± 0.5, F (1, 13) = 0.002 *p* = 0.96].

### 2.2. Pb Levels in Offspring Blood and Brain

As shown in Table 1, median blood Pb levels in offspring at post-natal day (pnd) 23 were 36 times higher than baseline (*p* < 0.01). Internal exposure to Pb translated into median Pb concentrations in cortex and hippocampus equal to 2.7 and 4.9 times the baseline (*p* < 0.01 and <0.001, respectively).

### 2.3. Neurodevelopmental Test Battery in Pup Rats

Pb similarly affected offspring of both sexes at neonatal stage. Pb exposure through pregnancy and lactation did not affect body weight and body length from pnd 4 to 12. At weaning Pb pups had similar body weight compared to Veh pups (Supplementary Figure S1A,B).

As for sensorimotor development, all pups showed a gradual decline across days in latency to righting reflex and negative geotaxis (Supplementary Figure S1C,D). Latency to righting on a surface in Pb pups was significantly shorter than in Veh pups at pnd 4 [*p* < 0.05, Pb × pnd interaction F (3, 30) = 3.681 *p* < 0.01].

**Figure 2:**
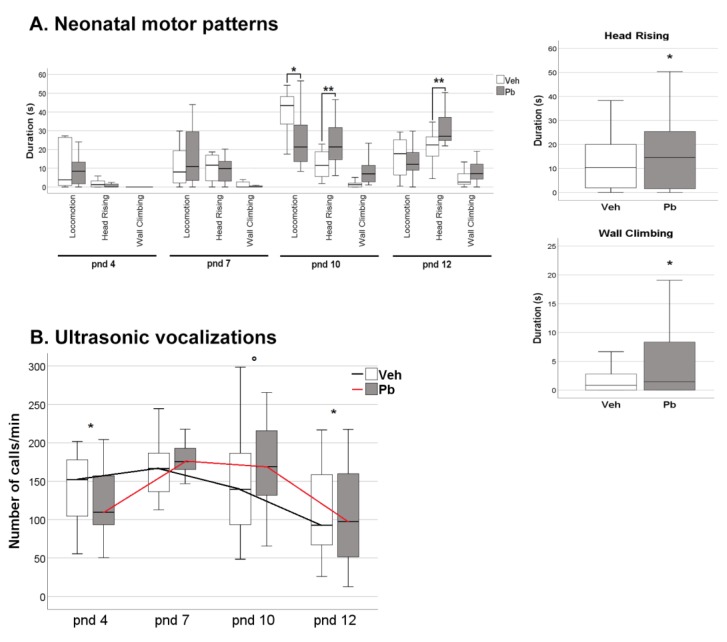
Effects of developmental Pb exposure on neonatal motor patterns and ultrasonic vocalizations (USVs) emitted by pups during a 3 min-test at post-natal day 4, 7, 10, and 12. (**A**) Duration of motor behaviors, namely locomotion, head rising and wall climbing, significantly affected in Pb pups of both sexes, * *p* < 0.05, ** *p* < 0.01; (**B**) Temporal profile of USV emission, * *p* < 0.05 vs. post-natal day 7, ° *p* < 0.05 vs. post-natal day 12. Data are sex-pooled represented; *n* = 12 (6 females and 6 males)/group.

The analysis of spontaneous movements indicated other Pb effects on selected motor patterns (Figure 2A), namely locomotion [*p* < 0.05, Pb × pnd interaction F (3, 30) = 2.963 *p* < 0.05], head rising [main effect of Pb F (1, 10) = 5.57 *p*< 0.05; Pb × pnd interaction F (3, 30) = 5.664 *p* < 0.01], and wall climbing [main effect of Pb F (1, 10) = 7.79 *p* < 0.01]. Pb pups spent less time in locomotion compared to Veh at pnd 10 in favor of head rising and wall climbing indicating a stereotyped/perseverative profile.

While displaying these spontaneous movements, Pb pups emitted a number of calls comparable to Veh, showing a similar temporal profile of emission, with peak of emission of USVs at pnd 7–10 (Figure 2B). Number of calls emitted by pups when separated from mother and siblings are indicative of early emotional and communication development.

On pnd 13 during the homing test, Pb and Veh pups took similar time to reach the nest arm and spent similar time there compared to Veh pups, indicating comparable olfactory discrimination and preference in the two groups (Supplementary Figure S2).

### 2.4. Behavioral Testing in Adolescent Rats

**Figure 3:**
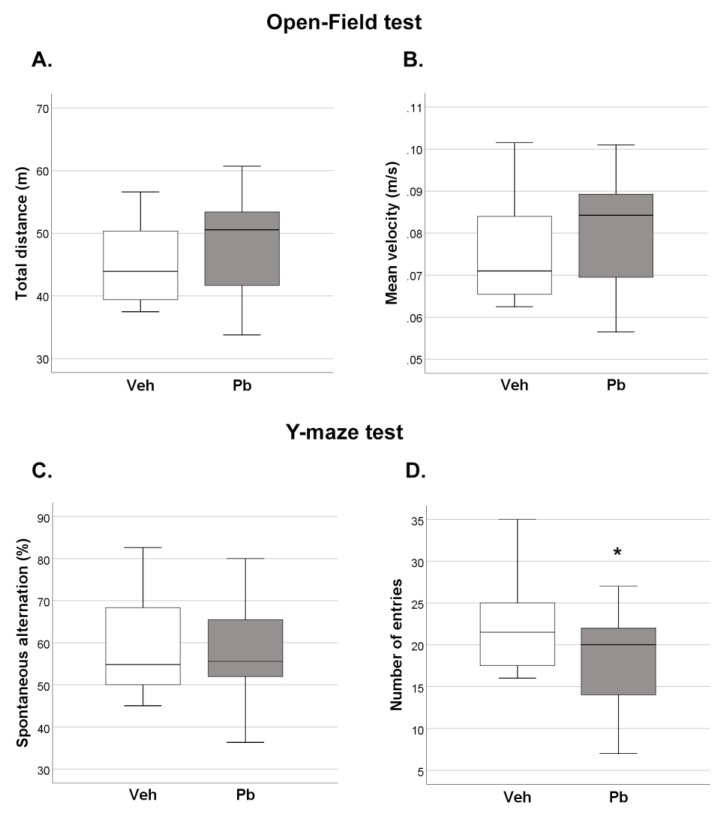
Effects of Developmental Pb Exposure on Locomotor Activity and Spontaneous Alternation Assessed in Open-Field (OF) and Y-Maze Test, Respectively. (**A**) Distance and (**B**) mean velocity in 10-min OF exploration; (**C**) Spontaneous alternation; and (**D**) number of arm entries in 5-min Y-maze test. Data are sex-pooled represented; *n*= 16 (7 females and 9 males)/group; * *p* < 0.05.

At adolescence, no Pb effects were found on locomotor activity (measured by total distance and mean velocity) exhibited during exploration of the novel environment (open-field test), Figure 3A,B. The mild motor effects evidenced in the first ten days of life disappeared after weaning, a recovery likely due to greater sensory and motor integration with age. Adolescent Pb rats also exhibited spatial working memory performance (measured by percentage of spontaneous alternation in Y-maze test) similar to Veh rats (Figure 3C). However, we detected a main effect of Pb on total number of Y-maze arm entries [F (1, 27) = 6.113 *p* = 0.02]: Pb rats performed less entries compared to Veh rats, suggesting decrement of explorative behavior (Figure 3D). Notably, Pb effects did not differ in the two sexes at adolescence.

### 2.5. Behavioral Testing in Adult Rats

**Figure 4:**
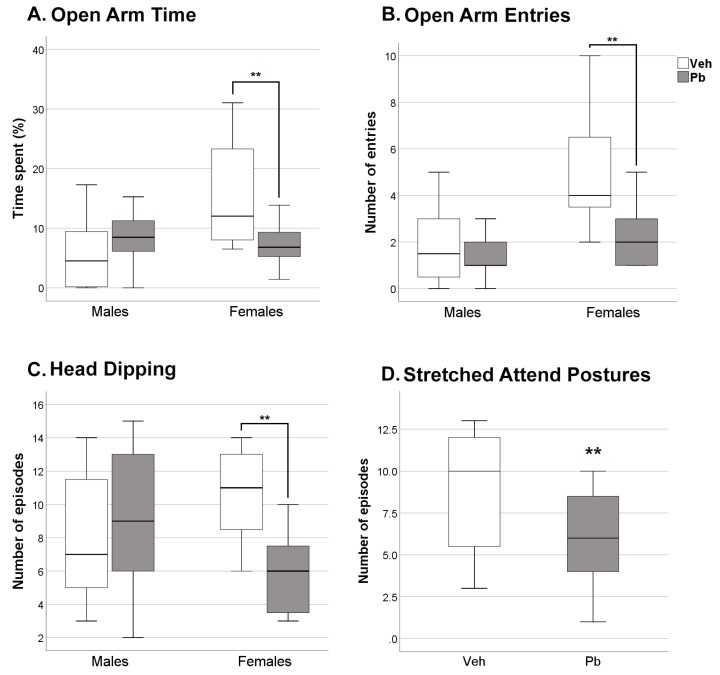
Effects of Developmental Pb Exposure on Anxiety-like Behavior Assessed in Elevated Plus Maze (EPM). Parameters affected selectively in female Pb rats in 5-min EPM, namely (**A**) time spent in open arms, (**B**) number of entries in open arms, (**C**) number of head dipping episodes; and (**D**) number of stretched-attend posture (SAP) episodes altered in Pb rats of both sexes (sex pooled value); *n* = 16 (7 females and 9 males)/group; ** *p* < 0.01.

As for anxiety-like behavior (measured in the Elevated Plus Maze, EPM) at adulthood, Pb effects were found selectively in female sex. Pb female rats visited the open arms with lower frequency compared to Veh female rats [*p* < 0.01, Pb × sex interaction F (1, 28) = 3.842 *p* = 0.06] and spent less time there [*p* < 0.01, Pb × sex interaction, F (1, 28) = 7.059 *p* = 0.01] exhibiting a more anxious profile (Figure 4A,B). In addition, Pb female rats performed a lower number of head dipping episodes (investigating the area beneath the EPM) compared to Veh female rats [*p* < 0.01, Pb × sex interaction F (1, 28) = 4.112 *p* = 0.05], Figure 4C. A main effect of Pb (both sexes) was found in stretched-attend postures (SAP, posture in which the body is stretched forward then retracted to the original position without any forward locomotion) frequency [F (1, 28) = 6.258 *p* = 0.01], a posture related to risk assessment behavior when approaching a novel area, with Pb rats exhibiting less SAP episodes than Veh rats (Figure 4D).

**Figure 5:**
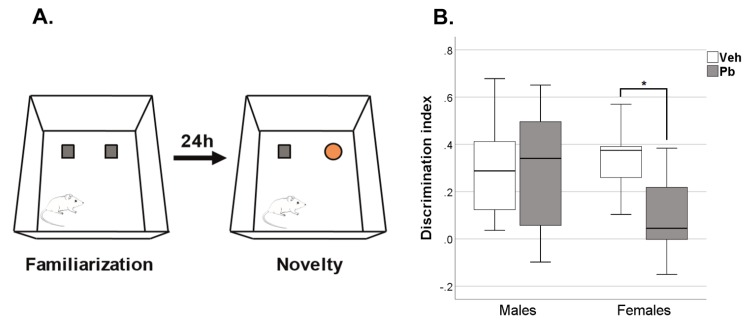
Effects of Developmental Pb Exposure on Long-Term Recognition Memory Assessed in Novel Object Recognition (NOR). (**A**) Schematic representation of the NOR task, consisting of Familiarization phase with two identical objects (represented as squares) and Novelty phase in which one of the two familiar objects (represented as a square) was replaced by a novel object (represented as a circle); (**B**) Discrimination index between a familiar object and novel one during Novelty session. *n* = 16 (7 females and 9 males)/group; * *p* < 0.05.

As for long-term recognition memory (measured in the Novel Object Recognition, NOR), Pb effect was found only in female rats [Z = −2.10, *p* < 0.05] that failed to discriminate between the novel object and the one previously explored (familiar), Figure 5.

**Figure 6:**
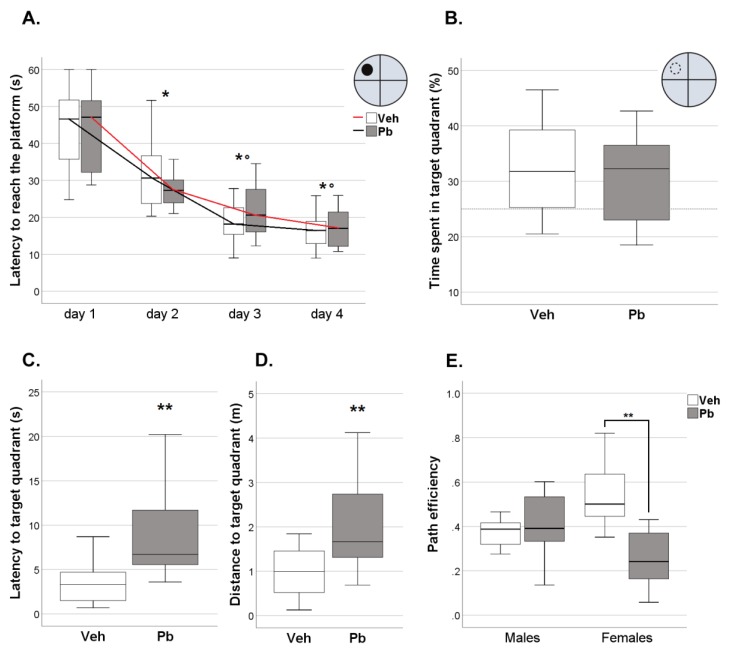
Effects of Developmental Pb Exposure on Spatial Memory Assessed in a Morris Water Maze (MWM). (**A**) Spatial learning across four days of training, * *p* < 0.05 vs. day 1, ° *p* < 0.05 vs. day 2; (**B**) Percentage of time spent in the target quadrant; parameters affected in Pb rats of both sexes during Probe trial, namely (**C**) Latency to reach the target quadrant and (**D**) Distance moved to reach the target quadrant; (**E**) Path efficiency in Probe trial selectively impaired in Pb female rats, ** *p* < 0.01. Data are sex-pooled represented, except for (E); *n* = 16 (7 females and 9 males)/group.

In spatial learning (measured in four days of training in a Morris Water Maze, MWM), Pb rats significantly decreased their escape latencies to reach the hidden platform across days in a similar fashion to Veh rats (Figure 6A). As for retention memory (measured by a single Probe trial 24 h after the last training trial), Pb rats spent significantly more time in the quadrant where the platform was located during training (target quadrant) than in the remaining quadrants, similarly to Veh rats (Figure 6B). Pb rats of both sexes, however, took significantly more time to reach the target quadrant [*Z* = −3.93, *p < 0*.01] and travelled longer distance [F (1, 28) = 14.029 *p* < 0.01] than Veh rats (Figure 6C,D). Analysis of path efficiency revealed a main effect of Pb in the female sex only [*Z* = −2.87, *p <* 0.01] with less efficient strategy shown by Pb female rats (Figure 6E).

### 2.6. Hippocampal NMDAR and AMPAR Evaluation

Organization of the glutamatergic response during development, to shape excitatory synaptic connectivity, depends both on the balanced expression of NMDAR and AMPAR subunits and their proper distribution between the synaptic and extra-synaptic site [26]. To evaluate whether in vivo developmental Pb exposure modulates both protein expression and insertion at the synapse of GluN2A, GluN2B, and GluN1 subunits of NMDAR and GluA1 and GluA2 subunits of AMPAR, hippocampi from male and female offspring at pnd 23 and 70 were homogenized and processed to obtain the Triton Insoluble Fraction (TIF) representative of the post-synapse [38]. To avoid any confounding factors, hippocampi were obtained from behavioral naïve animals.

**Figure 7:**
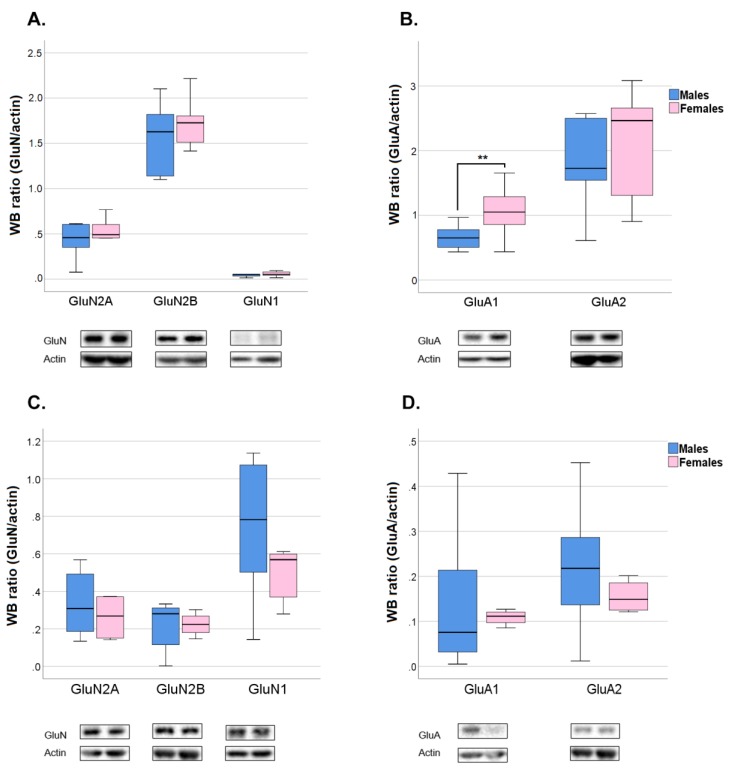
Sex Dimorphism in the Expression of N-methyl-D-Aspartate (NMDA) and α-amino-3-hydroxy-5-methyl-4-isoxazolepropionic acid (AMPA) Receptor Subunits in the Triton-Insoluble Fraction (TIF) of 23 and 70 Post-Natal Day Rat Hippocampi. Quantification and representative Western blots of glutamatergic NMDA and AMPA receptor subunits in male control and female control (Veh) rats at post-natal day 23 (**A**,**B**) and post-natal day 70 (**C**,**D**). Data were normalized on actin levels and are represented as ratio on actin (*n* = 5–7); ** *p* < 0.01.

NMDAR and AMPAR subunit total expression and amount at the post-synaptic site were not different between sexes, with the only exception of GluA1 subunit of AMPAR that was higher in female TIF at pnd 23 (Supplementary Table S1 and Figure 7).

**Figure 8:**
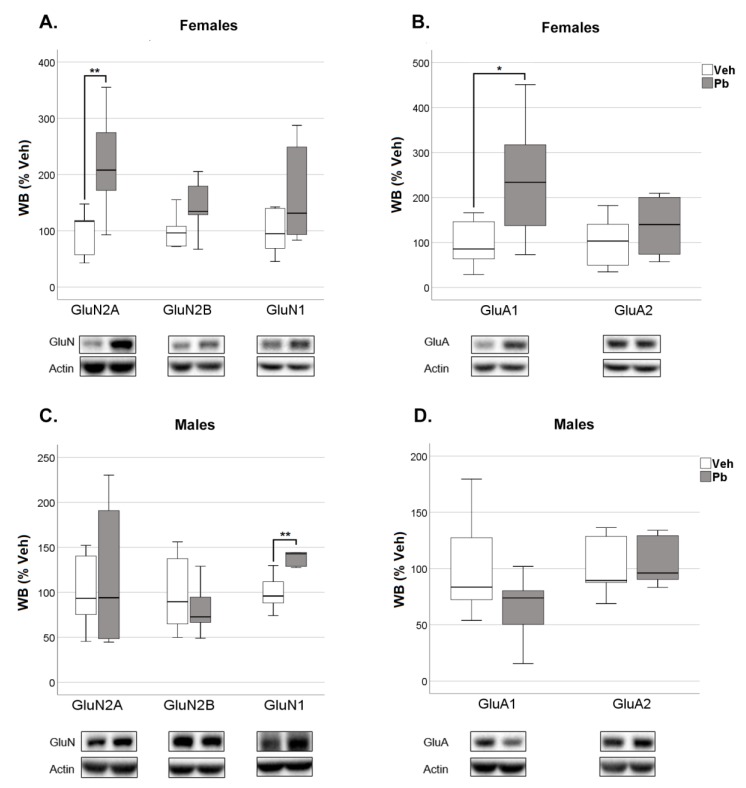
Effect of Developmental Pb Exposure on the Expression of NMDA and AMPA Receptor Subunits in the Triton-Insoluble Fraction (TIF) of 23 Post-Natal Day Female and Male Rat Hippocampi. Quantification and representative Western blots of glutamatergic receptor subunits in females (**A**,**B**) and males (**C**,**D**) in Veh and Pb rats. Data were normalized on actin levels and are represented as % of Veh (*n* = 5–8); * *p* < 0.05, ** *p* < 0.01.

Exposure to Pb significantly increased GluN2A (Figure 8A) and GluA1 (Figure 8B) protein levels in female TIF compared to Veh, with no changes in total protein expression of GluN2A, GluN2B, and GluN1 of NMDARs and GluA1 and GluA2 of AMPARs in the homogenate of both pnd 23 female and male rat hippocampi (Supplementary Table S2). The effect was specific for female rats as no significant changes in the expression of the afore-mentioned subunits were observed in males TIF at pnd 23 (Figure 8D), although an increase in GluN1 occurred (Figure 8C).

**Figure 9:**
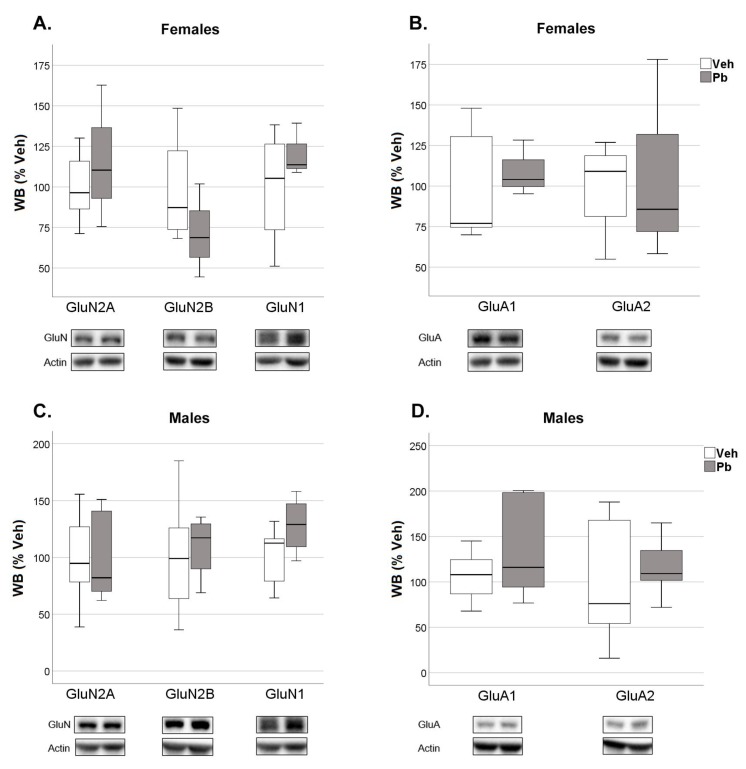
Effect of Developmental Pb Exposure on the Expression of NMDA and AMPA Receptor Subunits in the Triton-Insoluble Fraction (TIF) of 70 Post-Natal Day Female and Male Rat Hippocampi. Quantification and representative Western blots of glutamatergic receptor subunits in females (**A**,**B**) and males (**C**,**D**) in Veh and Pb rats. Data were normalized on actin levels and are represented as % of Veh (*n* = 3–7).

The molecular alterations observed at pnd 23 were completely restored and no other effect occurred after a 47 days withdrawal period from Pb. Thus, Pb did not affect both total and TIF expression of any of the considered subunits at pnd 70 (Supplementary Table S3 and Figure 9).

Finally, we focused our attention on Pb modulation of spine density, which is under the control of NMDAR subunits distribution at the synaptic spine [31,39] and represents a target for Pb [37,40,41]. Under our experimental conditions, no effect on PSD-95 expression was observed at pnd 23 and 70 (Supplementary Figure S3), suggesting the absence of spine loss.

## 3. Discussion

In this study, we aimed at evaluating the sex dependent vulnerability to 5 mg Pb/kg b.w. per day exposure during gestation and lactation in a rodent model, considering both behavioral outcomes and neurobiological correlates in a longitudinal perspective.

We observed: (i) behavioral changes induced by Pb exposure regardless of sex that include alterations in neonatal spontaneous movements and spatial memory retention (Figure 2 and Figure 6); (ii) behavioral deficits induced by Pb exposure in the female sex only, consisting in increased anxiety-like behavior (Figure 4), impairment in recognition memory (Figure 5) and spatial memory accuracy (Figure 6); and (iii) selective alterations in glutamatergic receptors distribution in the female sex (Figure 8).

The effects of Pb on early motor patterns have not been investigated in detail in previous rodent studies. They concern both sexes, and consist mainly in increased “stereotyped” explorative responses (head rising and wall climbing) at the expenses of locomotion, possibly mirroring the inability to inhibit inappropriate responding and perseveration described in both rodents and humans exposed to Pb [42].

As adults, Pb exposed rats acquire the spatial task at a rate comparable with that of Veh rats, but they show a mild deficit in spatial memory retention as revealed by the longer latency to reach the target quadrant during the probe trial. Spatial memory is a behavioral domain affected by developmental exposure to Pb: previous data collected in rats reported marked impairment in the MWM performance after early life exposure to Pb at exposure levels ranging from 150 to 2000 mg L^−1^ in drinking water [43,44,45,46,47,48], with the exception of one study in which 100 mg L^−1^ was administered [49]. The use of lower exposure levels explains the milder effects observed in our study, which, however, confirm that hippocampal-based spatial memory is a specific behavioral target of developmental Pb.

Sex differences became evident at adulthood. Two behavioral domains appear specifically sensitive to sex dependent vulnerability to Pb, namely anxiety and recognition memory. The anxiogenic effects of developmental exposure to Pb have been previously reported at doses higher than the one of the present study, without considering differences in the two sexes [50,51,52,53]. In the present study, adult female rats developmentally exposed to Pb show increased anxiety compared to Veh female rats when assessed in a task widely validated to measure anxiety responses, the EPM (Figure 4), specifically based on the natural aversion for open and unsafe spaces (the open arms) and preference for closed and protected spaces (the closed arms of the maze). Behavior in this task is normally sex-dimorphic: female rats spend more time in open arms of EPM and exhibit head dipping more frequently than male rats, displaying lower anxiety levels [54,55,56,57,58,59]. Here we report that Pb exposure blunts sex dimorphism as Pb female rats show a marked reduction of time spent in the open arms, their behavior being similar to that observed in males in this same task. Interestingly, similar blunting of sex-dimorphisms in anxiety responses were also observed following developmental exposure to a recognized endocrine disrupting chemical (EDC) such as bisphenol-A [60,61] as well as to the neurotoxic organophosphate chlorpyrifos [62,63].

Additionally, female rats exposed to Pb show a marked impairment in long-term memory. More specifically, they failed to show the expected preference for the novel object compared to the familiar one in the NOR task (Figure 5) and displayed lower spatial accuracy in the memory retention of escape platform position (detected by path efficiency parameter) in MWM (Figure 6). To our knowledge, this is the first evidence of long-term effects of Pb on non-spatial declarative memory.

The present findings are consistent with studies in which Pb exposure through gestation and lactation led to impairment in learning and memory and depressive-like behavior in female but not male rats [36,43,45,46,64,65,66]. We cannot rule out the possibility that adolescent rats could also be affected, as our longitudinal experimental design required the application of a battery of tests covering different behavioral domains knowingly sensitive to detect potential Pb-induced changes in age-specific abilities. In such a methodological framework, we ran tests from least invasive (stressful) to most invasive to decrease the chance that behavioral responses are altered by prior to test history [67]. Spontaneous alternation is a task suited to the novelty-seeking profile of the adolescent rats and can detect impairment in working memory, being at the same time less requiring and stressful of the MWM. By choosing different tests at different life stages, we also avoided habituation bias due to repetition of the identical behavioral test in the same rat. Specifically, behavioral response could be modified by previous experience of experimental paradigm and apparatus (i.e., typical decrease of locomotor activity and anxiety-like behavior once the subject attains familiarity with the test environment).

The formation and maturation of developing glutamatergic circuitry is the result of modification in synapses as ruled by patterns of neuronal activity and modulation in the expression and trafficking of NMDA and AMPA receptors subtypes, which contribute to the molecular basis of learning and memories [31,39,68]. As such, NMDA and AMPA dependent synaptic plasticity is essential for learning and spatial representation [69,70]. We observed an increased expression of GluN2A and GluA1 subunits in female hippocampus at pnd 23 (Figure 8). This effect is limited to the post-synaptic site and it is not associated with differences in the expression of the subunits in the whole hippocampus (Supplementary Table S2). GluN2A, which is encoded by the gene GRIN2A, is among the NMDAR subunits that have been strongly implicated in Pb neurotoxicity [37,47,71,72,73,74].

Notably, polymorphic variants in GRIN2A leading to altered NR2A expression are associated with neuro-behavioral anomalies in children [34,75,76]. Our results reveal the ability of Pb to affect ionotropic glutamate receptors composition at the post-synapse in vivo, an effect so far investigated only in vitro [77]. The lack of altered total expression of both GluN2A and GluA1 suggests that Pb favors a redistribution of these two subunits into female hippocampal spines. In accordance with the observation that GluN2A-containing NMDARs support the trafficking of GluA1 [78], Pb increased levels of GluA1 at the synapse might be the consequence of the increased GluN2A. Investigating Pb effect on NMDAR activity in our experimental condition, would be relevant to support the possibility that Pb alter the polarity of synaptic modification in female hippocampus acting on the subunit composition of NMDARs. Most of the literature focusing on the Pb modulation of NMDA receptors subunits reports a decrease of total GluN2A mRNA and protein expression [37,71,72]. The discrepancy with our results might be due to different factors, or their combination, such as the higher concentration of Pb used (750 mg L^−1^), the observation obtained in both sexes with no distinction, the different rat strain (Long–Evans versus Sprague Dawley) and the examination of animals that had been previously handled in behavioral protocols. The last point is particularly relevant considering that exposure to behavioral test may influence gene expression in the hippocampus [79]. The molecular effects observed at pnd 23 were completely recovered in the adult age, in spite of persistent behavioral alterations. These results are in accordance with the observation that alterations on the NMDAR subunits induced by Pb during development tend to reduce with ageing [80]. Intriguingly, Pb effects on learning and memory and executive functions by variants of GRIN2A are stronger in a 7-year than a 2-year old children [76]. The delayed behavioral effects we observed in females might be due to the unbalance by Pb of NMDAR- mediated synaptic plasticity that rules the development of behavioral processes under hippocampal control. In addition, developmental Pb exposure induces persistent inhibition of neurogenesis and alters the pattern of differentiation of newly born cells in the dentate gyrus of rat hippocampus, which could, at least partly, account for the behavioral and cognitive impairment observed in adulthood [51]. Of note, anxiety-like behaviors have been highly correlated with hippocampal GluA1 expression. Lower hippocampal GluA1 levels predict longer times in open arms and shorter times in closed arms of the EPM suggesting that higher GluA1 levels are associated with increased anxiety [57], in accordance with our results.

Recent overviews have included Pb among EDCs, hypothesizing a direct interference with sex hormones throughout early development to explain its sex dependent effects [81,82,83,84]. However, mechanisms by which Pb promotes endocrine-related, sex specific effects on behavioral outcome are still undetermined. Further studies are necessary to explore the hypothesis that Pb at environmentally relevant doses directly interferes with hormonal or neuroendocrine pathways. To date, being brain and behavior sexually dimorphic in mammals [85,86] it can be expected that molecular pathways implicated in complex behaviors, as well as epigenetic mechanisms, may be differentially modulated by exogenous stressors in the two sexes [36,66,82,87,88,89]. Differences between sexes with regard to patterns of exposure, chemical absorption, and metabolism might be equally relevant and deserve specific attention in future experimental studies.

Overall, our behavioral and molecular data indicate more pronounced effects in females than males developmentally exposed to Pb, which by interfering with biochemical trajectories linked to the maturation of the excitatory circuits early in life may set the stage for behavioral alterations later in life. Of note, in epidemiological studies where sex effects were examined, adverse outcomes from developmental Pb exposures have been described more frequently in males than in females [35,36,90,91] in contrast to experimental findings. However, it has to be noted that the influence of sex/gender on outcomes may depend on the type of outcomes measured, the age of outcome assessment and, last but not least from the statistical methods applied. The interpretation of potential sex-specific effects across studies might be affected by differences in the statistical approach followed, namely including the sex in the model, separating analysis by sex, or considering the interaction between sex/gender and toxicant exposure [92].

Evidence derived from experimental models addressing the issue of sex differences in brain and behavior might enhance the attention of epidemiology towards the proper consideration of sex/gender dependent vulnerability in population studies.

## 4. Materials and Methods

### 4.1. Animals

Following the adaption period, female rats (Wistar strain, weighing 250 ± 25 g) were randomly assigned to one of the two experimental groups (Vehicle, Veh or Pb) to receive 0 or 50 mg L^−1^ Pb (corresponding to 5 mg Pb/kg body weight per day), respectively, via drinking water 4 weeks prior to breeding throughout pregnancy and lactation until weaning (pnd 23). Female rats were mated with males (2 females:1 male) for 4 to 5 days to cover the duration of an estrous cycle.

Day of birth was designated as pnd 0. On pnd 1, litter were culled to equal numbers to standardize litter size, with an aim to have ten pups, sex balanced, per litter. At pnd 1 the sex of all pups in each litter was attributed on the basis of anogenital distance.

At weaning (pnd 23) the sex of each pups was again checked to confirm correspondence with sex attribution at birth. Male and female offspring were separated and housed 4 per cage up to the end of all experiments. All the animals were kept under standard animal housing (temperature 20  ±  2 °C; humidity 60–70%) with food and water *ad libitum*, under a 12h-12h light/dark cycle (lights on from 7:00 a.m. till 7:00 p.m.).

All experimental procedures were carried out in accordance with the European and Italian legislation (2010/63/EU, Dl 26/2014) and were approved by the Ethical Committee (Organismo Preposto per il Benessere Animale, OPBA) of the Italian National Health Institute (Istituto Superiore di Sanità, ISS) and by the Italian Ministry of Health (specific authorization n° 843/2016-PR, 09/07/2016).

### 4.2. Pb Exposure

Lead acetate (Pb(Ac)_2_·3H_2_O) was purchased from Sigma Aldrich (Merck KGaA, Darmstadt, Germany). Test solutions for animal treatment were prepared in soft tap water, slightly acidified (pH 4.6) with acetic acid, and containing no Pb acetate (Veh) or Pb acetate at a concentration 50 mg L^−1^ of Pb (as element). The medium ensured complete dissolution of the Pb salt and acceptance by the animals was the same as plain tap water. The Pb concentration in the test solutions was analytically checked (0.0004 and 50.8 mg Pb L^−1^, respectively) and stability was verified for 5 days. The solutions were prepared afresh every 3–4 days and used as drinking water for animal treatment.

### 4.3. Behavioral Testing

Neonatal stage: one female and one male offspring from 6 litters for each experimental group (Veh: 6 females and 6 males; Pb: 6 females and 6 males) underwent the neurodevelopmental test battery described below, including somatic, motor and sensorial assessment on pnd 4, 7, 10, 12, and 13.

For identification purposes, on pnd 4 pups were tattooed on the paw with animal tattoo ink (Ketchum permanent Tattoo Inks green paste, Ketchum Manufacturing Inc., Brockville, ON, Canada).

Juvenile/adult stage: female and male rats (from 6 Veh and 9 Pb litters) not subjected to neurodevelopmental test battery, were assessed in the following behavioral test: Open-Field (OPF, pnd 30), Spontaneous alternation (Y-maze, pnd 35), Elevated Plus Maze (EPM, pnd 60), Novel Object Recognition (NOR, pnd 63–65) and Morris Water Maze (MWM, pnd 68–72). Number of animals undergoing each behavioral test are reported in Figure legends.

All apparatus were cleaned with 70% alcohol following each animal testing. All behavioral procedures were carried out between 9:00 a.m. and 3:00 p.m.

#### 4.3.1. Analysis of Ultrasonic Vocalizations and Spontaneous Movements in Isolated Pups

On each day of testing (pnd 4, 7, 10, and 12), a single pup was placed into an empty glass container (diameter 5 cm; height 10 cm), located inside a sound-attenuating Styrofoam box, and USVs were recorded during a 3-min test as described in [93]. Number of calls were analyzed by Avisoft SASLab Pro (Avisoft Bioacoustics, Berlin, Germany).

Concomitant with the USV recording, the spontaneous movements of the pups were also videotaped. In accordance with previous studies focused on neonatal rodent behavior [91], the following behavioral patterns were scored: locomotion (general translocation of the body of at least 1 cm in the glass container), immobility (no visible movement of the animal when placed with all the four paws on the floor), head rising (a single rising of the head up and forward), face washing (forepaws moving back and forth from the ears to the snout and mouth), wall climbing (alternating forelimb placing movements on the wall of the container), pivoting (locomotor activity involving the front limbs alone and resulting in laterally directed movements), and curling (roll, vigorous side-to-side rolling movements while on the back; curl, a convex arching of back while on side or back, bringing head in a closer opposition to hump/hindlimb region).

Frequency and duration of each behavioral pattern were analyzed by using The Observer XT software (Noldus, Wageningen, The Netherlands).

#### 4.3.2. Somatic Growth and Sensorimotor Development Assessment

At the end of the 3-min recording session, each pup was assessed for reflex development and somatic growth (body weight and length) from pnd 4 to 12, as previously described [93].

The righting reflex was assessed by placing the pup on its back over a flat surface: the time needed to return to the natural position (all four paws on the floor) was measured using a stopwatch. The reflex was tested once in each day of assessment with a cut-off latency of 60 s.

The negative geotaxis was assessed by placing the pup on a 30-cm incline plane (20°), in a head down position. The time required to reorient to a head up position was recorded using a stopwatch and the cut-off time was 60 s.

#### 4.3.3. Homing Test

To assess early discriminative performances and maternal preference behavior, on pnd 13, pups were separated from the dam and kept for 30 min in an incubator (Elmed Ginevri 0GB 1000, Roma, Italy) at 28 ± 1 °C. The apparatus consisted in a grey Plexiglas T-shaped maze (start arm: 25 × 9 cm; choice arms: 12.5 × 9 cm; height: 8.5 cm). Each pup was gently placed in the start arm and allowed to freely explore the maze for 3 min. The time taken by the pup to reach the goal arm (containing nest litter), number of entries and time spent in the three arms were recorded and analyzed by The Observer XT software (Noldus, Wageningen, The Netherlands).

#### 4.3.4. Open-Field (OF)

To assess the locomotor activity during the exploration of novel environment, rats were tested in the OF test. The OF apparatus consisted of a black Plexiglas box (80 × 80 × 60 cm). Each subject was scored individually after being placed in one corner of the apparatus and spontaneous locomotor activity of the animals were video-recorded for 10 min (as described in [94]). Distance travelled and mean velocity were analyzed using ANY-Maze software (Stoelting Europe, Dublin, Ireland).

#### 4.3.5. Y-Maze

To assess the spatial working memory and explorative activity, rats were tested in the Y-maze test. The apparatus consisted of three identical arms (50 × 16 × 32 cm) diverging at 120° angle one to the other and an equilateral triangular central area. Each animal was placed in the center of the Y-maze and was free to explore the arena for 5 min. The following dependent variables were registered and analyzed by The Observer XT software (Noldus, Wageningen, The Netherlands): total number of arm entries and a sequential list of arms entered to assess number of alternations. An arm entry was scored when rat placed the four paws within that arm. An alternation was defined as an entry into three different arms on consecutive choices. Spontaneous alternation was calculated using the following formula: (number of alterations/total number of entries − 2) × 100.

#### 4.3.6. Elevated Plus Maze (EPM)

To assess the anxiety-like behavior, rats were tested in the EPM based on the natural conflict between the exploration of new areas and avoidance of unsafe areas. The EPM comprised two open arms and two closed arms that extended from a common central platform. The apparatus was constructed from Plexiglas (black floor, clear walls) and elevated to a height of 60 cm above the floor level. Rats were individually placed on the central platform facing a closed arm and allowed to freely explore the maze for 5 min (as described in [94]). Conventional measures were the frequencies of total, open and closed entries (arm entry = all four paws into an arm) and time spent in each arm. Ethological measures included frequency and duration scores for wall rearing, immobility, grooming, head dipping (exploratory movement of head/shoulders over the side of maze) and stretched-attend postures (SAP, exploratory posture in which the body is stretched forward and then retracted to the original position without any forward locomotion). Parameters were analyzed by The Observer XT software (Noldus, Wageningen, The Netherlands).

#### 4.3.7. Novel Object Recognition (NOR)

In order to evaluate the recognition memory of one previously explored object (familiar) compared with one novel object, rats were tested for the NOR test. NOR was performed in OF apparatus (a black Plexiglas box, 80 × 80 × 60 cm). Rats were tested during three sessions (habituation, familiarization and novelty/retention) separated by an interval of 24 h. In the familiarization session (duration 10 min), each rat was faced with two identical objects placed in two adjacent corners (20 cm from the walls) and the time exploring freely each object was recorded. In the retention trial (duration 10 min), one of the two familiar objects was replaced by a novel object and the time exploring each was recorded. Exploration time (computed when the snout pointed to the object at a distance ≤1cm) was scored by The Observer XT software (Noldus, Wageningen, The Netherlands). Discrimination index score was calculated using the following formula: (tn − tf)/(tn + tf), where tn—the amount of time rats explored the novel object and tf—the amount of time rats explored the familiar object.

#### 4.3.8. Morris Water Maze (MWM)

To assess the spatial learning and memory rats were tested in the MWM test. MWM was performed in a circular black pool (diameter 210 cm) filled with 24 °C water rendered opaque by the addition of atoxic acrylic paint (Giotto, Pero, Italy). An escape platform (diameter 10 cm) was submerged 2 cm below the water level. Animals used distal visual-spatial cues (posters on the walls of the room) to find the hidden escape platform located in the center of the target quadrant.

Each rat underwent to two phases of MWM: a spatial learning phase (training) of four days duration followed by a single Probe trial 24 h after the last training trial.

##### Training

During the spatial learning phase (four days with four consecutive trials per day) rats were released into the pool from one of the four starting points (one per each quadrant) and trained to find the hidden escape platform. The order of the sequence was changed pseudo-randomly between days. A trial was finished when the animal found the escape platform or when 60 s had elapsed. If a rat failed to find the platform, it was gently guided to the platform by the experimenter. Rats remained on the platform for 30 s. After each trial rats were dried and when the session finished they were returned to their colony room.

##### Probe Trial

Twenty four hour after training, spatial memory was assessed with a probe trial in which the escape platform was removed and rats were released from the quadrant opposite to target quadrant (which contained the escape platform during the training phase) and allowed to swim for 60 s in searching for it.

Latency to reach the escape platform, time spent in each quadrant, latency and distance moved to first entry to target quadrant and path efficiency were recorded and analyzed by ANY-Maze software (Stoelting Europe, Dublin, Ireland).

### 4.4. Blood and Brain Pb Determinations

Blood and brain dissection (cortex and hippocampus) were obtained from rats sacrificed at pnd 23; blood samples were collected in EDTA (0.2 mg/100 mL) from the left atrium of the heart of rats anesthetized by isoflurane. Samples were stored at −80 °C.

Sample treatment was performed in a clean room laboratory. A Milli-Q Element System (Millipore, Molsheim, France) was used to obtain high purity water for sample preparation. Nitric acid (HNO_3_, Carlo Erba Reagenti, Rodano, Italy) and H_2_O_2_ (Sigma-Aldrich, Merck KGaA, Darmstadt, Germany) were of ultrapure grade. The Pb calibration standard and internal standard (Rh) were prepared from certified standards solutions of 1000 ± 3 µg mL^−1^ (High Purity Standard, Charleston, SC, USA). Blood was diluted 1:50 with 0.2% *v/v* HNO_3_ and 0.005% *v/v* Triton X-100 (J.T. Baker). Procedural blanks submitted to the same sample handing of blood specimens were prepared and their concentration subtracted from that of study samples. Tissues were digested by microwave irradiation with HNO_3_ and H_2_O_2_ by means of an Ultrawave closed-vessel system equipped with temperature and pressure control (Milestone, FKV, Bergamo, Italy). An 8800 Triple Quad mass spectrometer from Agilent Technologies (Tokyo, Japan) was used for ICP-MS determinations. The standard addition method was used for quantification. Accuracy was checked by the control material Seronorm Whole Blood L1 (measured value 15.0 ± 0.5 µg L^−1^ vs. certified value/range of acceptable values 14.8/13.8–15.8 µg L^−1^), for blood, and the certified reference material BCR 8414 Bovine Muscle (measured value 368 ± 15 ng g^−1^ vs. certified value/range of acceptable values 390/366–414 ng g^−1^), for tissues.

### 4.5. Hippocampal Tissues Processing

At pnd 23 and 70, behaviorally naïve animals of both sexes were euthanized by decapitation and hippocampi were collected and stored at −80 °C.

The homogenate and the post-synaptic Triton-insoluble fraction (TIF) were prepared from hippocampal tissue as previously described in [95]. Briefly, tissues were homogenized with a Teflon-glass potter in ice-cold lysis buffer (sucrose 0.32 M, Hepes 1 mM, MgCl_2_ 1 mM, NaHCO_3_ 1 mM and PMSF 0.1 mM, at pH 7.4) in presence of a complete set of proteases (Roche Diagnostics, Basel, Switzerland) and phosphatases inhibitors (Sigma-Aldrich, Merck KGaA, Darmstadt, Germany). An aliquot of the homogenate was collected and immediately frozen in dry ice: this fraction represents the homogenate sample (HOMO). The HOMO was then centrifuged at 13,000x *g* for 15 min. The resulting supernatant was removed and the pellet was resuspended in ice-cold buffer (75 mM KCl, 1% Triton-X 100) and centrifuged at 100,000× *g* for 1 h. The supernatant was discarded and the pellet obtained was homogenized in a glass-glass potter in 20 mM ice-cold Hepes and then stored at −80 °C until processing.

### 4.6. Western Blotting

Protein content of HOMO and TIF’s samples was quantified using Bio-Rad dye reagent protein assay (Hercules, CA, USA). After measuring protein concentration, all samples were standardized at 1 μg/uL concentration and 15 μg of protein per sample were loaded in each lane of the gel. After gel electrophoresis, proteins were transferred to a nitrocellulose membrane (Bio-Rad, Hercules, CA, USA) and blocked for 2 h at room temperature with I-block solution (Applied Biosystems, Foster City, CA, USA) before antibody incubation. Western blot analysis were performed using antibodies raised against NMDA glutamate receptor subunits GluN2A, GluN2B and GluN1, and AMPA glutamate receptor subunits GluA1, GluA2 diluted 1:1000, and actin diluted 1:2500. Secondary antibodies were diluted 1:10,000 and all dilutions were made in I-block solution. Quantification of Western blotting analysis was performed by means of computer-assisted imaging (Image Lab Software from Bio-Rad) after normalization on actin levels. All the reagents were purchased from Sigma-Aldrich (Merck KGaA, Darmstadt, Germany). Monoclonal GluN2B and GluA2 antibodies were purchased from NeuroMab (Davis, CA, USA); polyclonal GluA1 from Calbiochem (Merck KgaA, Darmstadt, Germany); monoclonal GluN1 antibody from Invitrogen (Carlsbad, CA, USA); monoclonal GluN2A, actin and secondary anti-mouse antibodies from Sigma Aldrich (Merck KgaA, Darmstadt, Germany); and secondary anti-rabbit antibody from Bio-Rad (Hercules, CA, USA).

### 4.7. Statistical Analysis

Reproductive performances of dams exposed to Pb were presented as means +/− SD (standard deviations).

Biodistribution data were reported as medians and interquartile ranges (IQR: 75th–25th centile). Comparisons between Veh and Pb animals were carried out by the Mann–Whitney Wilcoxon test (two-tailed).

Behavioral data were analyzed by analysis of variance (ANOVA) with group and sex as between-factors and day/trial/quadrant as repeated measures followed by post-hoc Tukey’s analysis on significant interaction effects. When parametric assumptions were not fully met, the non-parametric Mann–Whitney *U*-test was used.

For the protein expression data, statistical analysis between Veh and Pb groups was performed, within each sex, by using unpaired Student’s t-test.

Values deviating from the mean by more than 2.5 times the standard deviation (SD) were discarded as considered outliers (cut off = 2.5 SD). A 95% level (*p* < 0.05) was considered statistically significant.

## 5. Conclusions

Developmental Pb exposure (through gestation and lactation) affects selected behavioral domains and glutamatergic receptor expression and distribution in a sex-dependent fashion. Here we investigated one of several molecular mechanisms potentially underlying the behavioral abnormalities observed, providing the basis for further insights such as glutamatergic receptors functional studies. Overall, our behavioral and biochemical data indicate more pronounced effects in females than males exposed to Pb during development. We suggest that Pb may set the stage for altered behavior later in life by interfering with biochemical trajectories linked to early maturation of the excitatory circuits.

## Figures and Tables

**Table 1:** Pb concentrations in blood (µg/mL) and brain tissues (µg/g) of offspring at post-natal day 23.

Tissue	Group	*N*	Median (IQR)
Blood	Veh	4	0.007 (0.01)
	Pb	4	0.255 (0.05) **
Cortex	Veh	4	0.165 (0.07)
	Pb	6	0.452 (0.19) **
Hippocampus	Veh	4	0.141 (0.01)
	Pb	6	0.685 (0.46) ***

** *p* < 0.01, *** *p* < 0.001 vs. Veh group.

## Supplementary Materials

Supplementary Materials can be found at https://www.mdpi.com/1422-0067/21/8/2664/s1. Supplementary Figure S1. Effects of Developmental Pb Exposure on Somatic Growth and Sensorimotor Development. (A) Body weight; (B) Body length; (C) Righting reflex; and (D) Negative geotaxis. Data are sex-pooled and represented as mean ± SEM; *n*= 12 (6 females and 6 males)/group; * *p* < 0.05. Supplementary Figure S2. Effects of Developmental Pb Exposure on Nest Odor Recognition Assessed in Homing Test at Post-Natal Day 13. (A) Latency to reach the nest arm and (B) Time spent in each arm of T-maze. Data are sex-pooled and represented as mean ± SEM. *n* = 10–12/group. Supplementary Figure S3. Effect of Developmental Pb Exposure on the Expression of PSD95 in the Total Homogenate of 23 Post-Natal Day Male (A), Female (B), 70 Post-Natal Day Male (C), and Female (D) Rat Hippocampi. Quantification and representative Western blots of glutamatergic receptor subunits in Veh and Pb rats. Data were normalized on actin levels. Values are represented as % of Veh (mean ± SEM, *n* = 5–7). Supplementary Table S1. Sex Dimorphism on the Expression of NMDA and AMPA Receptor Subunits in the Total Homogenate of 23- (A) and 70-Post-Natal Day (B) Veh rats. Data were normalized on actin levels. Values are represented as mean ± SEM and n; ns—not statistically significant. Supplementary Table S2. Effect of Developmental Pb Exposure on the Expression of NMDA and AMPA Receptor Subunits in the Total Homogenate of 23 Post-Natal Day Female (A) and Male (B) Rat Hippocampi. Data were normalized on actin levels. Values are represented as % of Veh (mean ± SEM and n); ns—not statistically significant. Supplementary Table S3. Effect of Developmental Pb Exposure on the Expression of NMDA and AMPA Receptor Subunits in the Total Homogenate of 70 Post-Natal Day Female (A) and Male (B) Rat Hippocampi. Data were normalized on actin levels. Values are represented as % of Veh (mean ± SEM and n); ns—not statistically significant.
