# Supplementary material for: Sex-Dependent Effects of Developmental Lead Exposure in Wistar Rats: Evidence from Behavioral and Molecular Correlates
Source: Int J Mol Sci. 2020 Apr 11;21(8):2664. doi: 10.3390/ijms21082664 (PMC7216048; doi:10.3390/ijms21082664)
Supplement: Supplementary file 1 [file ijms-21-02664-s001.zip › Legends_SupplementaryFigureTable.docx]

Supplementary Figure 1. Effects of developmental Pb exposure on somatic growth and sensorimotor development. (A) Body weight; (B) Body length; (C) Righting reflex; (D) Negative geotaxis.

Data are sex-pooled and represented as mean ± SEM; n= 12 (6 females and 6 males)/group; * p<0.05

Supplementary Figure 2. Effects of developmental Pb exposure on nest odor recognition assessed in Homing test at post-natal day 13. (A) Latency to reach the nest arm; (B) Time spent in each arm of T-maze. Data are sex-pooled and represented as mean ± SEM. n= 10-12/group.

Supplementary Figure 3. Effect of developmental Pb exposure on the expression of PSD95 in the HOMO fraction of 23 post-natal day (A) and female (B) and 70 post-natal day (C) and female (D) rat hippocampi. Quantification and representative western blots of glutamatergic receptor subunits in Veh and Pb rats. Data were normalized on actin levels. Values are represented as % of CTRL (mean ± SEM, n=5-7).

Supplementary Table 1. Sex dimorphism on the expression of NMDA and AMPA receptor subunits in the total hippocampal homogenate of 23- (A) and 70- post-natal day (B) control rats. Data were normalized on actin levels. Values are represented as mean ± SEM and n; ns = not statistically significant.

Supplementary Table 2. Effect of developmental Pb exposure on the expression of NMDA and AMPA receptor subunits in the total homogenate (HOMO) of 23 post-natal day female (A) and male (B) rat hippocampi. Data were normalized on actin levels. Values are represented as % of CTRL (mean ± SEM and n); ns = not statistically significant.

Supplementary Table 3. Effect of developmental Pb exposure on the expression of NMDA and AMPA receptor subunits in the total homogenate of 70 post-natal day female (A) and male (B) rat hippocampi. Data were normalized on actin levels. Values are represented as % of CTRL (mean ± SEM and n); ns = not statistically significant.
