# Supplementary material for: Sex-Dependent Effects of Developmental Lead Exposure in Wistar Rats: Evidence from Behavioral and Molecular Correlates
Source: Int J Mol Sci. 2020 Apr 11;21(8):2664. doi: 10.3390/ijms21082664 (PMC7216048; doi:10.3390/ijms21082664)
Supplement: Supplementary file 1 [file ijms-21-02664-s001.zip › SupplementaryTable1.pdf]

A. pnd 23

		MALE	FEMALE	
NMDA	GluN2A	0.438 ± 0.07<br>n = 6	0.592 ± 0.088<br>n = 7	ns
	GluN2B	0.409 ± 0.085<br>n = 5	0.448 ± 0.095<br>n = 5	ns
	GluN1	0.374 ± 0.084<br>n = 6	0.48 ± 0.170<br>n = 6	ns
AMPA	GluA1	0.483 ± 0.092<br>n = 6	0.495 ± 0.094<br>n = 6	ns
	GluA2	0.598 ± 0.145<br>n = 7	0.527 ± 0.103<br>n = 7	ns

B. pnd 70

		MALE	FEMALE	
NMDA	GluN2A	0.343 ± 0.029<br>n = 7	0.323 ± 0.022<br>n = 4	ns
	GluN2B	0.163 ± 0.035<br>n = 8	0.061 ± 0.011<br>n = 3	ns
	GluN1	0.18 ± 0.032<br>n = 8	0.283 ± 0.019<br>n = 5	ns
AMPA	GluA1	0.218 ± 0.033<br>n = 7	0.208 ± 0.017<br>n = 4	ns
	GluA2	0.245 ± 0.053<br>n = 7	0.157 ± 0.047<br>n = 5	ns
