# Supplementary material for: Sex-Dependent Effects of Developmental Lead Exposure in Wistar Rats: Evidence from Behavioral and Molecular Correlates
Source: Int J Mol Sci. 2020 Apr 11;21(8):2664. doi: 10.3390/ijms21082664 (PMC7216048; doi:10.3390/ijms21082664)
Supplement: Supplementary file 1 [file ijms-21-02664-s001.zip › SupplementaryTable2.pdf]

A. FEMALES

		CTRL	Pb 100 ppm	
NMDA	GluN2A	100 ± 25.85<br>n = 7	149.14 ± 38.51<br>n = 8	ns
	GluN2B	100 ± 10.75<br>n = 7	123.50 ± 42.36<br>n = 8	ns
	GluN1	100 ± 7.06<br>n = 7	97.96 ± 4.86<br>n = 8	ns
AMPA	GluA1	100 ± 21.70<br>n = 7	128.22 ± 18.74<br>n = 7	ns
	GluA2	100 ± 12.72<br>n = 7	91.66 ± 11.32<br>n = 8	ns

B. MALES

		CTRL	Pb 100 ppm	
NMDA	GluN2A	100 ± 13.20<br>n = 7	121.81 ± 16.18<br>n = 5	ns
	GluN2B	100 ± 20.19<br>n = 7	116.97 ± 13.18<br>n = 5	ns
	GluN1	100 ± 4.76<br>n = 6	100.46 ± 10.96<br>n = 4	ns
AMPA	GluA1	100 ± 16.45<br>n = 7	146.39 ± 17.66<br>n = 6	ns
	GluA2	100 ± 19.46<br>n = 6	110.42 ± 6.79<br>n = 5	ns
