# Supplementary material for: Sex-Dependent Effects of Developmental Lead Exposure in Wistar Rats: Evidence from Behavioral and Molecular Correlates
Source: Int J Mol Sci. 2020 Apr 11;21(8):2664. doi: 10.3390/ijms21082664 (PMC7216048; doi:10.3390/ijms21082664)
Supplement: Supplementary file 1 [file ijms-21-02664-s001.zip › SupplementaryTable3.pdf]

A. FEMALES

		CTRL	Pb 100 ppm	
NMDA	GluN2A	100 ± 10.57<br>n = 4	94.27 ± 13.06<br>n = 3	ns
	GluN2B	100 ± 22.67<br>n = 5	99.31 ± 29.37<br>n = 3	ns
	GluN1	100 ± 14.35<br>n = 4	99.82 ± 16.07<br>n = 3	ns
AMPA	GluA1	100 ± 10.71<br>n = 4	117.66 ± 23.54<br>n = 3	ns
	GluA2	100 ± 25.65<br>n = 4	160.49 ± 78.20<br>n = 3	ns

B. MALES

		CTRL	Pb 100 ppm	
NMDA	GluN2A	100 ± 4.77<br>n = 7	92.11 ± 6.37<br>n = 4	ns
	GluN2B	100 ± 8.50<br>n = 7	107.32 ± 13.71<br>n = 5	ns
	GluN1	100 ± 4.21<br>n = 6	124.47 ± 17.24<br>n = 4	ns
AMPA	GluA1	100 ± 9.35<br>n = 7	104.51 ± 4.98<br>n = 4	ns
	GluA2	100 ± 8.86<br>n = 8	121.87 ± 13.31<br>n = 4	ns
