# Supplementary figures and images for: Sex-Dependent Effects of Developmental Lead Exposure in Wistar Rats: Evidence from Behavioral and Molecular Correlates
Source: Int J Mol Sci. 2020 Apr 11;21(8):2664. doi: 10.3390/ijms21082664 (PMC7216048; doi:10.3390/ijms21082664)

## Somatic growth

**A.**

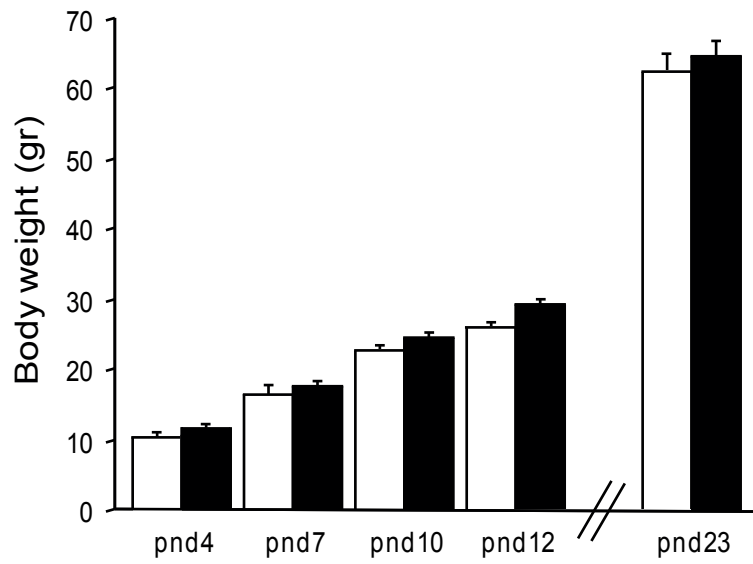

**B.**

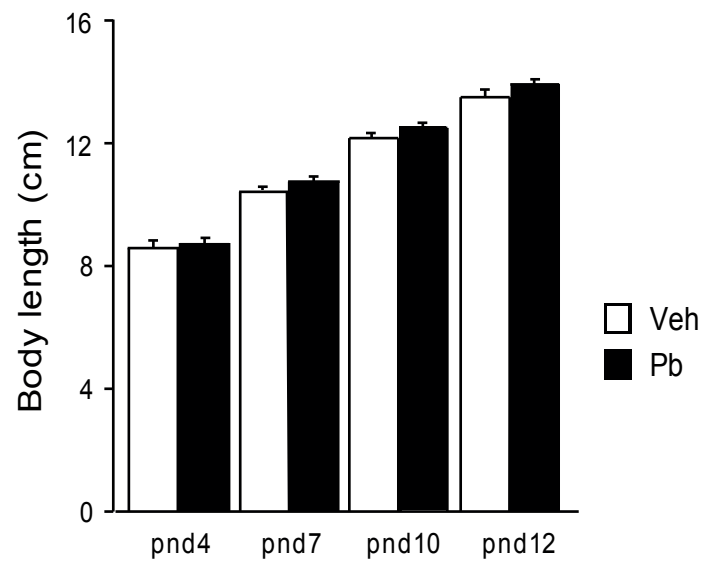

## Sensorimotor development

**C.**

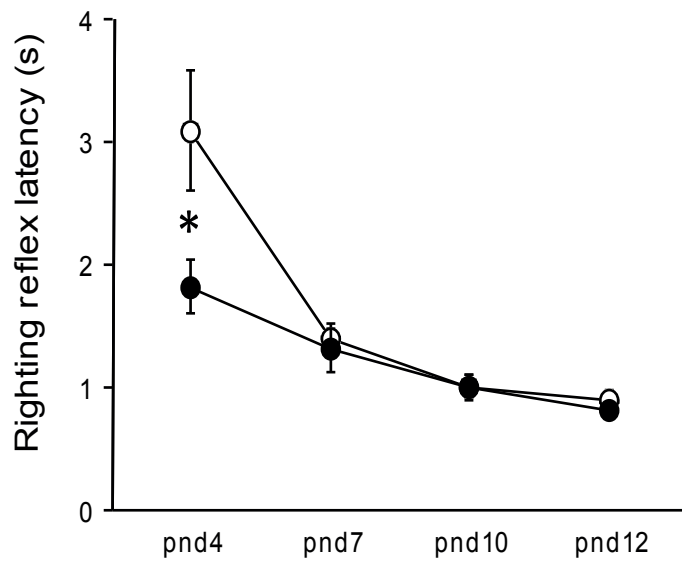

**D.**

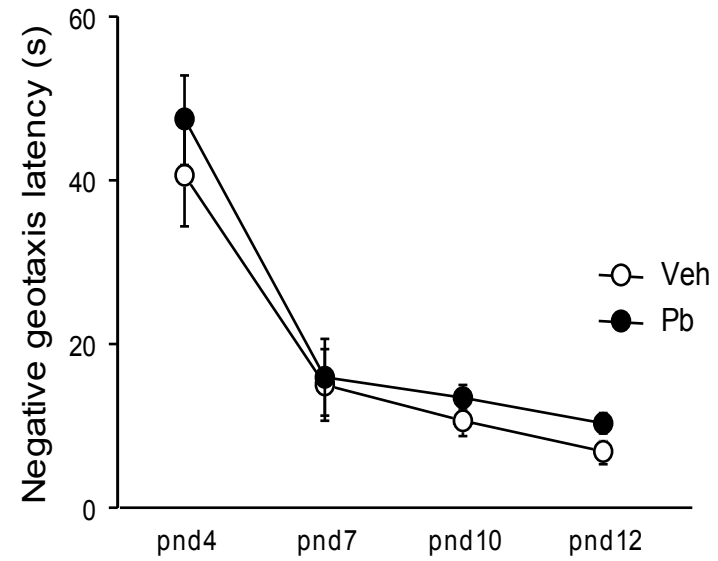

Supplement: Supplementary file 1 [file ijms-21-02664-s001.zip › SupplementaryFigure1.pdf]

**A.**

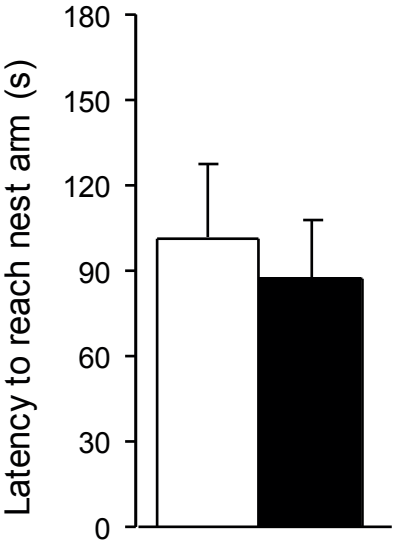

**B.**

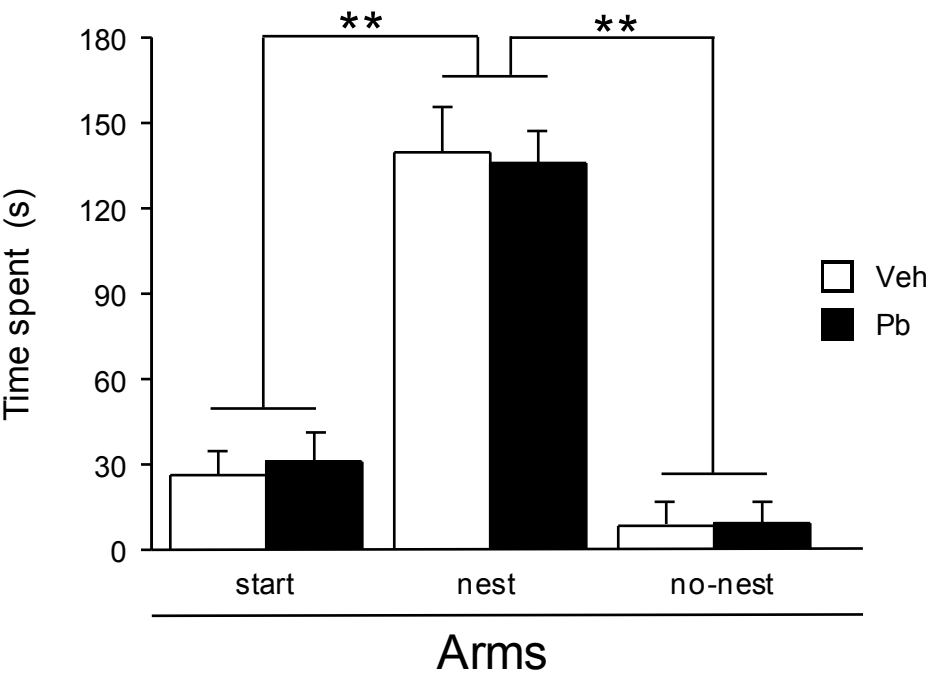

Supplement: Supplementary file 1 [file ijms-21-02664-s001.zip › SupplementaryFigure2.pdf]

**A.**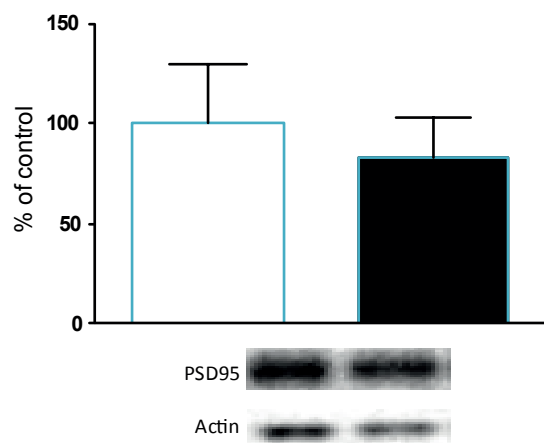**B.**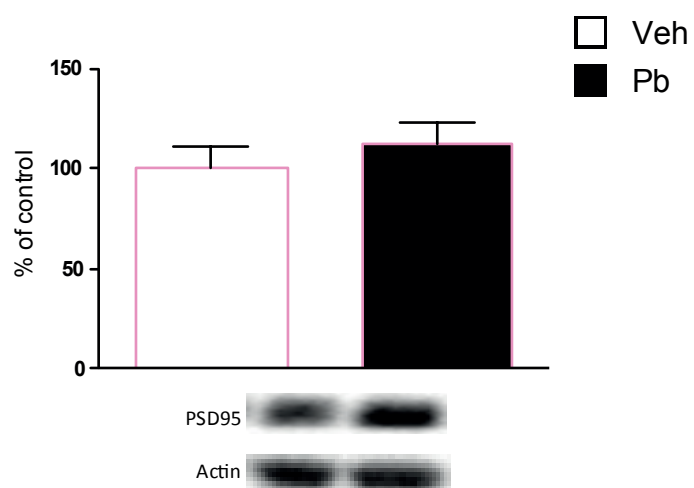**C.**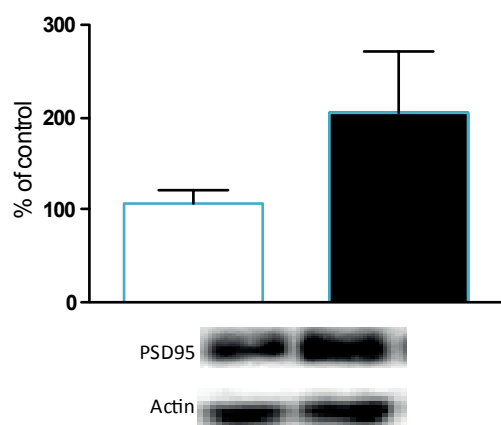**D.**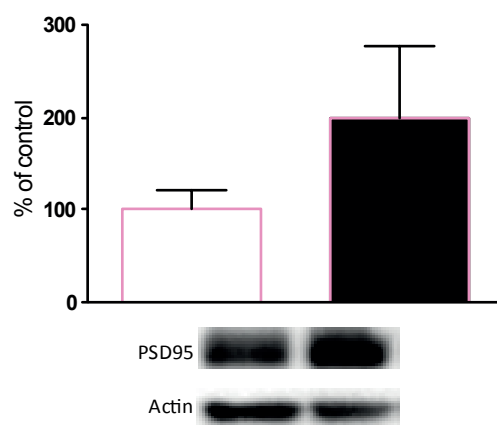

Supplement: Supplementary file 1 [file ijms-21-02664-s001.zip › SupplementaryFigure3.pdf]
